# Single-cell gene expression analysis of cryopreserved equine bronchoalveolar cells

**DOI:** 10.3389/fimmu.2022.929922

**Published:** 2022-08-23

**Authors:** Sophie E. Sage, Pamela Nicholson, Laureen M. Peters, Tosso Leeb, Vidhya Jagannathan, Vinzenz Gerber

**Affiliations:** ^1^ Swiss Institute of Equine Medicine, Department of Clinical Veterinary Medicine, Vetsuisse Faculty, University of Bern, Bern, Switzerland; ^2^ Next Generation Sequencing Platform, University of Bern, Bern, Switzerland; ^3^ Clinical Diagnostic Laboratory, Department of Clinical Veterinary Medicine, Vetsuisse Faculty, University of Bern, Bern, Switzerland; ^4^ Institute of Genetics, Vetsuisse Faculty, University of Bern, Bern, Switzerland

**Keywords:** single-cell mRNA sequencing, cell cryopreservation, equine respiratory system, equine immunology, cell annotation

## Abstract

The transcriptomic profile of a cell population can now be studied at the cellular level using single-cell mRNA sequencing (scRNA-seq). This novel technique provides the unprecedented opportunity to explore the cellular composition of the bronchoalveolar lavage fluid (BALF) of the horse, a species for which cell type markers are poorly described. Here, scRNA-seq technology was applied to cryopreserved equine BALF cells. Analysis of 4,631 cells isolated from three asthmatic horses in remission identified 16 cell clusters belonging to six major cell types: monocytes/macrophages, T cells, B/plasma cells, dendritic cells, neutrophils and mast cells. Higher resolution analysis of the constituents of the major immune cell populations allowed deep annotation of monocytes/macrophages, T cells and B/plasma cells. A significantly higher lymphocyte/macrophage ratio was detected with scRNA-seq compared to conventional cytological differential cell count. For the first time in horses, we detected a transcriptomic signature consistent with monocyte-lymphocyte complexes. Our findings indicate that scRNA-seq technology is applicable to cryopreserved equine BALF cells, allowing the identification of its major (cytologically differentiated) populations as well as previously unexplored T cell and macrophage subpopulations. Single-cell gene expression analysis has the potential to facilitate understanding of the immunological mechanisms at play in respiratory disorders of the horse, such as equine asthma.

## 1 Introduction

Single-cell sequencing technologies have brought a fresh impetus to biological research. With single-cell mRNA sequencing (scRNA-seq), it is now possible to study global gene expression at the single cell level in many complex tissues and heterogeneous cell populations. ScRNA-seq is a powerful tool for the generation of novel hypotheses. The applications of single-cell transcriptomics across the biomedical fields range from the identification of new cell types to the exploration of disease-specific pathological processes, unveiling novel biomarkers and potential therapeutic targets (1). Recently, scRNA-seq gave invaluable insights into the pathological mechanisms leading to respiratory decompensation in SARS-CoV-2 patients (2–4).

Lower airway diseases are common in horses and have a major impact on the equine industry and animal welfare. The processes at play in their pathogenesis are still ill defined due to their complexity, but also due to technical limitations. For the characterization of equine asthma, the most prevalent of these disorders, cytological examination of bronchoalveolar lavage fluid (BALF) is the most widely used technique in both clinical and experimental settings. It is, however, inherently subjective and only allows for the differentiation of five distinct leukocyte populations: macrophages, lymphocytes, neutrophils, mast cells and eosinophils (5). Antibody-based techniques such as immunohistochemistry and flow cytometry permit differentiation of further subpopulations (e.g. within lymphocytes), but in the horse they are restricted to a few cell types due to the limited pool of validated antibodies (6, 7). Individual mRNA transcripts can be measured in BALF mainly by RT-PCR to investigate the influence of various factors on the regulation of specific genes (8–11). This hypothesis-driven approach suffers from a low throughput and a significant investigator bias. In contrast, global transcriptomics is an unbiased, high throughput technique. However, critical differences between individual cells are obscured when performing bulk RNA sequencing of mixed cell populations. This is an important limitation knowing that the cellular composition of the lower respiratory tract, particularly when assessed by BALF sampling, is substantially affected by health status (12). ScRNA-seq enables the description of the different cell populations present in a sample concurrently with their individual transcriptome. Moreover, cell types can be identified without *a priori* knowledge of marker genes.

Single-cell gene expression analysis of bronchoalveolar cells has been successfully performed in humans (2–4), mice (13), ferrets (14) and dogs (15), but not in horses. Before scRNA-seq can be applied to the study of equine respiratory diseases, it is crucial to demonstrate its feasibility on BALF and to build an equine lung-specific reference database. Published scRNA-seq experiments on BALF use fresh samples to optimize cell viability and to avoid the transcriptional changes that could be associated with storage and processing. Being able to store equine BALF cells before scRNA-seq would have several advantages including reducing potential batch effects and facilitating large-scale longitudinal studies.

Here, we applied scRNA-seq to cryopreserved equine BALF cells as a proof of concept, with the aim to differentiate and characterize cell populations based on their transcriptional signatures. It should be noted that the potential effect of cryopreservation on gene expression was not assessed in this study.

## 2 Material and methods

### 2.1 Ethics statement

All animal experiments were performed according to the local regulations. This study was approved by the Animal Experimentation Committee of the Canton of Bern, Switzerland (BE07/19).

### 2.2 Study population

**Figure 1:**
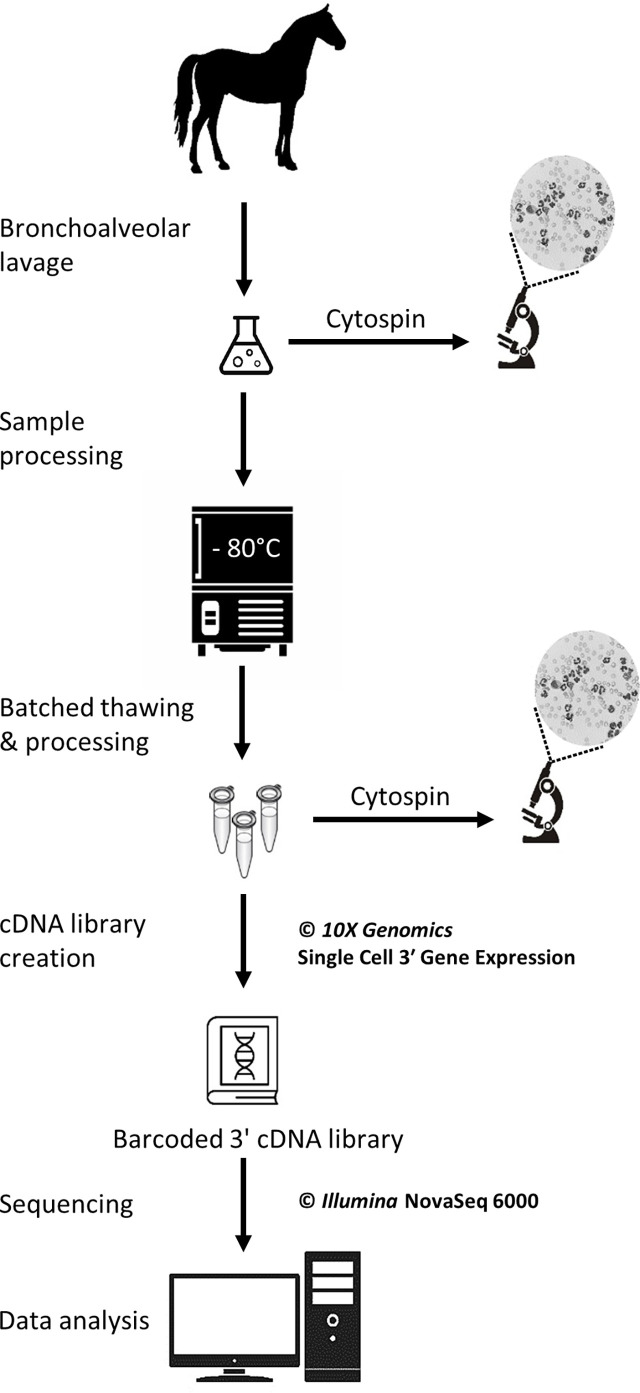
Experimental workflow for the single-cell mRNA sequencing of cryopreserved equine bronchoalveolar cells.

The study was carried out in February 2020. Three horses belonging to the university teaching herd were included. Characteristics of the study population are listed in **Table 1**. A standard physical examination was performed to assess systemic health. These horses suffered from mild-to-moderate equine asthma, but were in clinical remission at the time of the study. No medication other than alpha-2 agonists (sedation for teaching purposes) were administered for at least a month before the experiment. Lower airway inflammation status was assessed *via* clinical scoring (16), bronchoscopy and BALF cytology. The experimental workflow is summarized in **Figure 1**.

**Table 1:** Characteristics of the study population.

	Horse 1	Horse 2	Horse 3
Sex	Gelding	Gelding	Gelding
Age (years)	11	12	10
Breed	Trotteur Français	Franches-Montagnes	Selle Français
HOARSI	3	3	3
Clinical score (/23)	0	0	0
Tracheal mucus score (/5)	1	0	2
BALF yield* (%)	11	8	16

HOARSI, Horse Owner Assessed Respiratory Signs Index; BALF, Bronchoalveolar Lavage Fluid.

*BALF yield % = (Volume saline re-aspirated (mL)/Volume saline instilled (mL)) x 100.

### 2.3 Sample collection

A bronchoalveolar lavage was performed with endoscopic guidance under light sedation. Briefly, a flexible endoscope (VET-OR1200HD, Medical Solution GMBH, Wil, SG, Switzerland) was inserted into the pharynx *via* the nasal passages and passed down into the trachea. The endoscope was then advanced into the lower airways *via* the right mainstem bronchus until it wedged against a distal bronchus. Sterile 0.9% NaCl (250 mL) was instilled through the endoscope channel using 60-mL syringes. The fluid was then re-aspirated and the endoscope pulled out. The syringes’ content was pooled in a cooled silicone-coated glass bottle. The BALF was then filtered through a 40-µm cell strainer (BD Falcon™, Biosciences, USA, cat.352340) and kept on ice until processing.

### 2.4 Cytology

Cytocentrifuge preparations of the BALF were prepared within 30 minutes following collection. Briefly, BALF was centrifuged at 600 rpm for 8 minutes in a cytocentrifuge (Tharmac Cellspin^®^ I), and slides were subsequently stained with Wright-Giemsa stain (Hematek Stain Pak, Siemens Healthineers, Erlangen, Germany) on an automated slide stainer (Hematek 3000, Siemens Healthineers, Erlangen, Germany). The cell suspensions later obtained after sample thawing (sample used for scRNA-seq) were similarly processed. A manual differential cell count (macrophages, lymphocytes, neutrophils, eosinophils and mast cells) was performed on the cytocentrifuge preparations of the stained BALF and cell suspension using a minimum of 400 cells and 4 different microscopic fields at 1000x magnification with oil immersion.

### 2.5 ScRNA-seq

#### 2.5.1 Cryopreservation

The protocol used to freeze and subsequently thaw the BALF cells was adapted from a 10X Genomics protocol (CG00039 Rev D) intended for human peripheral blood mononuclear cells (PBMCs). The detailed laboratory protocol can be found in the **Supplementary material**. Samples were kept on ice throughout the freezing protocol. Cell count and viability were determined with a Moxi GO II™ cell counter (Witec AG, Sursee, LU, Switzerland) using propidium iodide 5:1000. The BALF was initially centrifuged at 300 rcf for 5 minutes at 4°C and the supernatant was removed. Cells were resuspended in RPMI (Gibco™ RPMI 1640 cat. 11875093) containing 40% fetal bovine serum (Gibco™ FBS cat.16000044) and 1 U/µL RNAse inhibitor (Roche^®^ Protector RNase Inhibitor) to achieve a concentration of 20x10^6^ cells/mL. An equivalent volume of freezing medium was added to achieve a concentration of 10x10^6^ cells/mL. The freezing medium consisted of RPMI (Gibco™ RPMI 1640 cat.11875093) with 30% dimethylsulfoxid (MP Biomedicals DMSO ≥99% cat.0219141880) and 40% fetal bovine serum (Gibco™ FBS cat.16000044) added. Cell suspension aliquots were dispensed into cryovials. The cryovials were placed into a pre-cooled cell freezing container (Corning™ CoolCell™ cat. 432005) at -80°C for 4 hours, before being transferred into a cryobox for storage at -80°C. The timespan between BALF collection and freezing was less than 2 hours for all samples.

#### 2.5.2 Thawing and resuspension

Samples were stored at -80°C for 5 to 6 days before thawing and further processing for scRNA-seq. Cryovials (one 1-mL aliquot per horse) were rapidly thawed in a water bath at 37°C. Cells were resuspended 5 times by incremental 1:1 volume addition of complete growth medium, which consisted of RPMI (Gibco™ RPMI 1640 cat.11875093) with 10% fetal bovine serum (Gibco™ FBS cat.16000044). Cells were centrifuged at 300 rcf for 5 min at room temperature. The supernatant was removed except for 1 mL, in which the cells were resuspended. Complete growth medium was added to achieve a total volume of approximately 10 mL. The cell concentration and viability were determined as previously described. A volume of suspension containing 6x10^5^ cells was transferred into a new tube and centrifuged 5 min at 300 rcf at room temperature. The supernatant was removed and the cells were resuspended into 400 µL resuspension solution, which consisted of phosphate-buffered saline (Gibco™ DPBS cat.14190094) containing 0.04% bovine serum albumin (Invitrogen™ UltraPure™ BSA cat.AM2616) and 0.8 U/µL RNAse inhibitor (Roche^®^ Protector RNase Inhibitor). The centrifugation step was repeated and the supernatant discarded. Resuspension solution was added with the goal of achieving a cell concentration between 700 and 1,200 cells/µL. Final cell concentration and viability were measured as described above. Cells were kept on ice until loading into the Chromium™ Controller (10X Genomics, Pleasanton, CA, USA). About 200 µL of the final cell suspension were used to prepare the cytospin slides (see section 2.4).

#### 2.5.3 Single-cell cDNA library preparation and scRNA-seq

GEM generation & barcoding, reverse transcription, cDNA amplification and 3’ gene expression library generation steps were all performed according to the Chromium Next GEM Single Cell 3’ Reagent Kits v3 User Guide (10x Genomics CG000383 Rev C) with all stipulated 10x Genomics reagents. Nuclease-free water was added to the cell suspensions to reach a total volume of 46.6 µL each, for a targeted cell recovery of 5,000 cells (see details in **Supplementary Table 1**). GEM generation was followed by a GEM-reverse transcription incubation, a clean-up step and 12 cycles of cDNA amplification. The resulting cDNA was evaluated for quantity and quality using a Thermo Fisher Scientific Qubit 4.0 fluorometer with the Qubit dsDNA HS Assay Kit (Thermo Fisher Scientific, Q32851) and an Advanced Analytical Fragment Analyzer System using a Fragment Analyzer NGS Fragment Kit (Agilent, DNF-473), respectively. Thereafter, 3ʹ gene expression libraries were constructed using a sample index PCR step of 15 cycles. Later, using the same double stranded cDNA, dual indexed libraries were also constructed using a library preparation kit and dual Index kit TT Set (10x Genomics part numbers 1000190 and 300041, respectively). These new libraries were constructed due to the dual indexing upgrade and the upgrade in NovaSeq 6000 reagent kits from v1 to v1.5 and they were generated following the relevant parts of 10 x Genomics User Guide CG000315. The aim of this upgrade in library type was to ensure that this pilot study is more compatible with future data. Any generated cDNA libraries were tested for quantity and size using fluorometry and capillary electrophoresis as described above. The cDNA libraries were pooled and sequenced with a loading concentration of 300 pM, paired end and either single or dual indexed, on an Illumina NovaSeq 6000 sequencer using a shared NovaSeq 6000 S1 Reagent Kit v1.0 (100 cycles; Illumina, 20012865) or a S4 Reagent Kit v1.5 (200 cycles; Illumina, 20028313). The quality of the sequencing runs were assessed using Illumina Sequencing Analysis Viewer (Illumina version 2.4.7) and all base call files were demultiplexed and converted into FASTQ files using Illumina bcl2fastq conversion software v2.20. At least 50,000 reads/cell were generated for each sample. All steps were performed at the Next Generation Sequencing Platform, University of Bern.

### 2.6 Computational analysis

**Figure 2:**
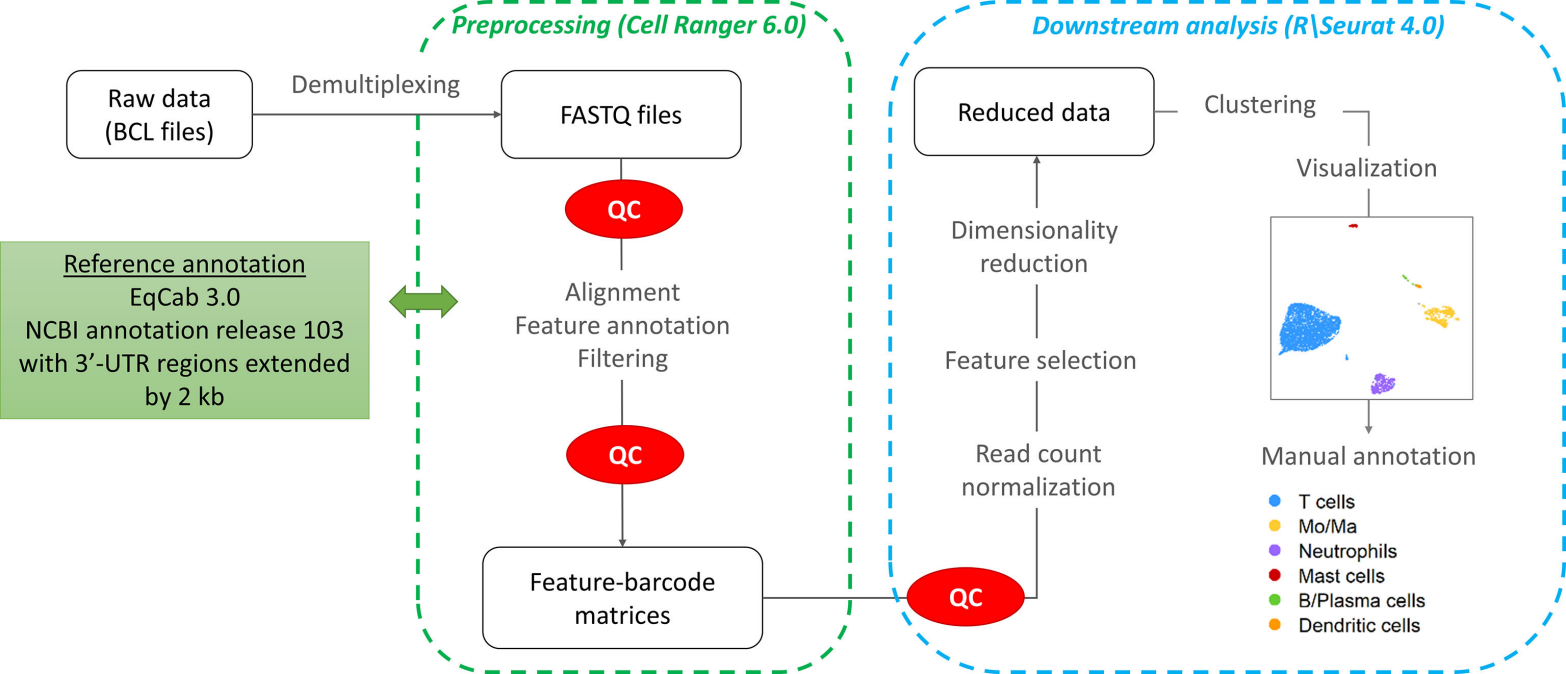
scRNA-seq data analysis workflow.

The workflow followed for the computational analysis is illustrated in **Figure 2**.

#### 2.6.1 Pre-processing

Raw sequencing data (*fastq* files) were converted to a count matrix of gene expression values using the Cell Ranger (v6.0) standard workflow. The annotations for 3’-untranslated regions of the genes in the reference genome (Equus caballus NCBI annotation release 103) were extended by 2 kb using a custom Python script and manual curation. All transcripts were extended except when the extension overlapped a neighboring gene. The summary metrics of the detected cells are provided in **Table 2**.

**Table 2:** Summary metrics of the detected cells for each sample.

	Horse 1	Horse 2	Horse 3
Estimated number of cells	1,460	1,931	2,017
Fraction reads in cells	86.0%	91.1%	78.8%
Mean reads per cell	115,356	71,580	88,570
Median UMIs per cell	2,922	2,637	2,376
Median genes per cell	1,340	1,256	1,061
Total genes detected	18,287	18,910	18,893
Sequencing saturation	88.7%	81.1%	84.5%
Reads mapped confidently to genome	92.5%	92.6%	91.7%
Reads mapped confidently to exonic regions	57.4%	58.6%	57.0%
Reads mapped confidently to transcriptome	53.6%	55.0%	57.0%

Data generated with CellRanger v6.0 using EqCab 3.0 NCBI annotation release 103 with 3’-UTR regions extended by 2 kb.

#### 2.6.2 Quality control and data normalization

Quality control and downstream analysis were carried out using the R software package Seurat (v4.0) (17). Based on visual data inspection, cells that contained less than 200 genes or more than 6,500 gene features and/or greater than 15% mitochondrial genes were filtered (**Supplementary Figure 1**). The raw data were normalized using global scale normalization.

#### 2.6.3 Principal component analysis (PCA) and cell clustering

After variance stabilizing transformation, the 2,000 most variable features were selected for dimension reduction and clustering. Dimensionality reduction was conducted using Principal Component Analysis (PCA). The number of principal components (PCs) was chosen based on an elbow plot. Clustering was performed on the first 16 PCs using the default Louvain algorithm (“FindNeighbors” function in Seurat). The optimal clustering resolution was chosen by visualizing the granularity with the “clustree” R package. The “FindClusters” function (Seurat) was used with a clustering resolution of 1.0. The clusters were visualized with the Uniform Manifold Approximation and Projection method (UMAP). After data integration, cluster visualization was only marginally improved. Cluster membership remained the same, except for a very small proportion of cells (0.5%) which were assigned to Mo/Ma instead of DC. Hence we elected to perform the downstream analysis without integration to avoid introduction of bias due to non-linear data integration approaches (18).

In order to better distinguish cell subpopulations, we independently reanalyzed three of the major cell populations using the “subset” function in Seurat. All previous steps were repeated. We selected 13 PCs with a 1.2 clustering resolution for monocyte/macrophages, 11 PCs with a 0.5 clustering resolution for T cells, and 8 PCs with a 0.7 clustering resolution for B/plasma cells.

#### 2.6.4 Cell cycle analysis

To investigate whether the cell cycle stage affected clustering, we used the “CellCycleScoring” function (Seurat package). Briefly, the lists of human markers for the G2M phase and the S phase (“*cc.genes.updated.2019”* from Seurat) were converted to their equine orthologs using the Biomart R package. Cells were divided into cycling (G2M phase) or resting (S phase) based on the score obtained.

#### 2.6.5 Cell cluster annotation

The cell clusters were annotated based on the expression of canonical markers and subsequently merged into major cell types. Cell cluster annotation was confirmed by subjective analysis of the list of markers identified with the “FindAllMarkers” Seurat function, using an adjusted *P*-value <0.05 and an average log2 fold change > 0.25. Expression of specific cell type markers was visualized using the “DotPlot”, “VlnPlot” and “FeaturePlot” functions. Cell type specificity of the markers was evaluated based on the information provided on the Human Protein Atlas version 21.0 database^a^. To facilitate cell cluster annotation, expression scores for cell-specific group of genes were calculated using the Seurat function “AddModuleScore”. Gene expression scores of Mo/Ma 2, Mo/Ma 4 and T cells were compared using a Kruskall-Wallis test, with *P*-value < 0.05 considered statistically significant. The differentially expressed genes (DEGs) for each of the 16 clusters can be found in **Supplementary Table 2**.

## 3 Results

Three university-owned horses known to be affected by mild-to-moderate equine asthma were included. They were considered systemically healthy based on a complete physical examination. The diagnosis of mild-to-moderate equine asthma was confirmed based on the following three criteria: a Horse Owner Assessed Respiratory Signs Index (HOARSI) score of 3 (19, 20); BALF cytology values of >10% and <25% neutrophils, >5% mast cells or >5% eosinophils; absence of increased breathing effort at rest (12). The low clinical score and tracheal mucus score indicated that the horses were in clinical remission at the time of inclusion (16, 21). Key characteristics of the study population can be found in **Tables 1**, **3**.

**Table 3:** Differential cell counts (DCC) obtained with conventional cytology and single-cell RNA sequencing (scRNA-seq).

	Horse 1			Horse 2			Horse 3		
	BALF cytology	Cell suspension cytology	scRNA-seq	BALF cytology	Cell suspension cytology	scRNA-seq	BALF cytology	Cell suspension cytology	scRNA-seq
Lymphocytes %	55.8	60.8	85.3	40.5	39.5	74.7	39.5	39.5	71.3
Macrophages %	25.8	22.8	7.4	44.0	48.0	13.7	46.0	48.5	16.9
Neutrophils %	5.8	4.0	3.4	14.5	12.0	10.5	14.0	10.0	11.0
Mast cells %	12.8	12.3	3.8	1.0	0.5	1.1	0.5	2.0	0.8
Eosinophils %	0	0	0	0	0	0	0	0	0
Lymphocytes/Macrophages	2.2	2.7	11.5	0.9	0.8	5.5	0.9	0.8	4.2

BALF cytology was performed on a fresh BALF sample shortly after collection. Cell suspension cytology was performed on an aliquot of the thawed sample used for scRNA-seq. ScRNA-seq DCC was calculated based on the number of cells present in each of the six major cell populations listed, with B and T cell clusters counted as lymphocytes and DCs counted as macrophages.

**Figure 3:**
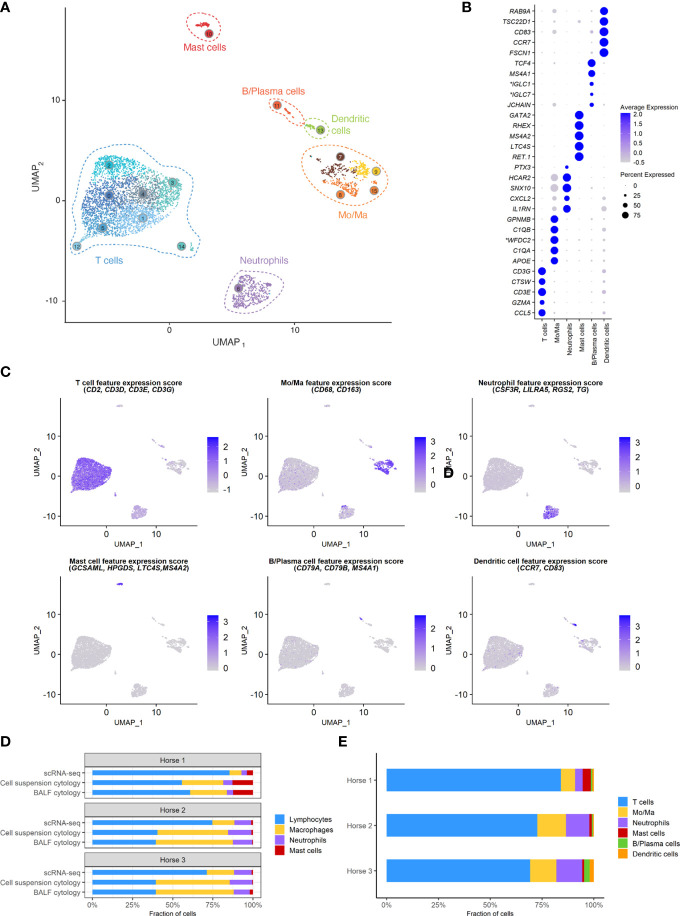
scRNA-seq analysis of 4,631 cryopreserved equine bronchoalveolar cells isolated from 3 horses. *Mo/Ma, monocytes/macrophages.* **(A)** The 16 clusters identified (indicated by numbers) are grouped into 6 major cell populations (UMAP visualization). **(B)** Dot plot of the 5 most upregulated genes in each major cell population. Dot size is proportional to the percentage of cells expressing the gene. Dot color intensity represents average gene expression. *Gene ID LOC102147726 annotated as Immunoglobulin Lambda-1 Light Chain (IGLC1), LOC100060608 as Immunoglobulin Lambda Constant 7 (IGLC7) and LOC102148710 as WAP four-disulfide core domain protein 2 (WFDC2) (NCBI EqCab3.0 v103). **(C)** Gene expression patterns used for cell type assignment. **(D)** Distributions of the major bronchoalveolar cell populations obtained with cytology on BALF, with cytology on the cell suspension (post cryopreservation) and with scRNA-seq on the cell suspension. T cells and B/plasma cells are counted as lymphocytes, while Mo/Ma and DCs are counted as macrophages. **(E)** Distribution of the major bronchoalveolar cell populations for each horse, based on scRNA-seq analysis.

A total of 5,408 cells were sequenced, of which 777 were filtered after quality control. Downstream analysis was thus performed on 4,631 cells. Unsupervised graph-based clustering produced 16 clusters, which were grouped into six major cell populations based on the expression of canonical markers (**Figure 3**). The differentially expressed genes (DEGs) for each of the 16 clusters can be found in (**Supplementary Figure 2**), Cells did not cluster based on the individual sample, therefore data integration was not performed. Clusters 7, 8, 9 and 15 expressing *CD163* and *CD68* were identified as monocytes/macrophages (Mo/Ma). Cluster 13 was annotated as dendritic cells (DCs) based on *CD83* and *CCR7* expression (22). These were most likely myeloid DCs, based on the upregulation of *FSCN1*, a gene not expressed in plasmacytoid DCs (23). Clusters 0, 1, 2, 3, 4, 5, 12 and 14 were annotated as T cells based on *CD2, CD3D, CD3E* and *CD3G* expression. Cluster 11 was annotated as B/Plasma cells based on *MS4A1*, *CD79A* and *CD79B* expression. Cluster 6 was annotated as neutrophils based on *TG*, *RGS2*, *LILRA5* and *CSF3R* expression. Lastly, cluster 10 was identified as mast cells based on *LTC4S*, *HPGDS*, *GCSAML* and *MS4A2* expression. Ribosomal protein (RP) genes were highly expressed in T cells and B/plasma cells (**Supplementary Figure 2**). After merging the clusters into these six major cell populations, the (DEGs) for each population were extracted (**Supplementary Table 3**). Analysis of these markers supported our annotations.

### 3.1 Cell distribution

The distribution of the six major cell populations identified with scRNA-seq was similar for the three horses, except for an increased mast cell proportion in horse 1 (**Figure 3E**). Next, we inspected the distribution of the five cytologically distinguishable leukocyte types (macrophages, lymphocytes, neutrophils, eosinophils and mast cells). We considered that under light microscopy, T cells and B/plasma cells are counted as lymphocytes, while Mo/Ma and DCs are counted as macrophages. To assess the effect of sample processing and cryopreservation on differential cell counts (DCC), we first compared the cytological DCC performed on fresh BALF to the cytological DCC performed on the final cell suspension. The cell distribution was mostly unchanged, suggesting cryopreservation did not significantly affect DCC. Next, we compared the cytological DCC of the cell suspension to the DCC calculated with scRNA-seq. The lymphocyte/macrophage ratio was found to be markedly higher with scRNA-seq compared to cytology. The proportions of the other cell populations were similar (**Figure 3D** and **Table 3**).

### 3.2 Composition of the monocytes-macrophages population

**Figure 4:**
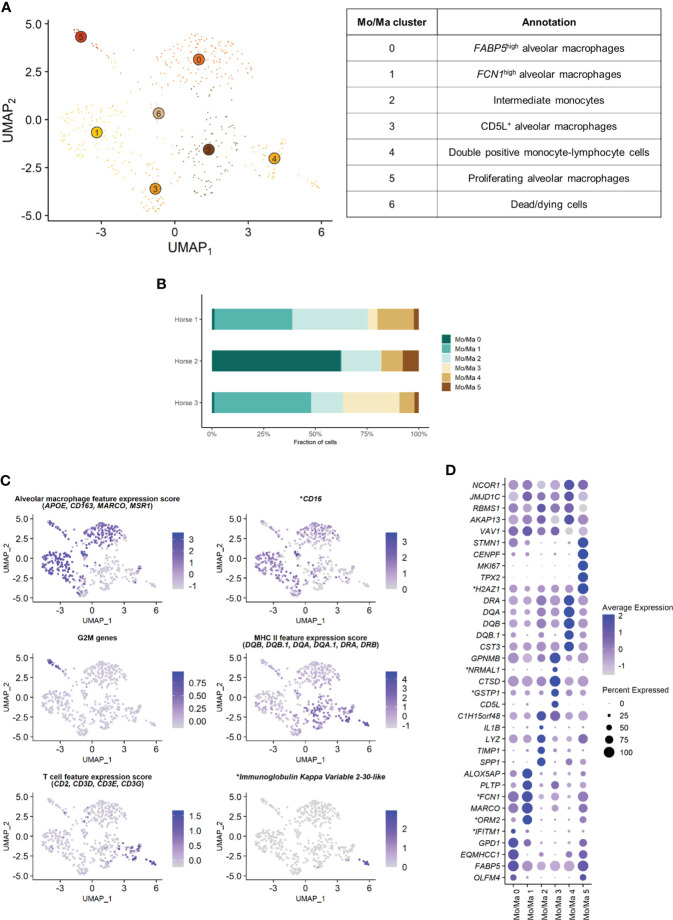
Independent analysis of the monocytes-macrophages (Mo/Ma) population (n=529 cells) **(A)** UMAP visualization of the 7 Mo/Ma clusters identified, with suggested cluster annotation. **(B)** Distribution of the Mo/Ma clusters for each horse, based on scRNA-seq analysis. **(C)** Gene expression patterns used for cell subtype assignment. *LOC100051526 annotated as CD16 (NCBI EqCab3.0 v103). **(D)** Dot plot of the 5 most upregulated genes in each cluster. Dot size is proportional to the percentage of cells expressing the gene. Dot color intensity represents average gene expression. Mo/Ma 6 (dead cells) is excluded. *LOC100146489 annotated as H2A.Z variant histone 1 (H2AZ1), LOC100067916 as NmrA like redox sensor 1 (NRMAL1), LOC100059533 as glutathione S-transferase pi 1 (GSTP1), LOC100069029 as ficolin 1 (FCN1), LOC100050560 as interferon induced transmembrane protein 1 (IFITM1) and LOC100050100 as orosomucoid 2 (ORM2) (NCBI EqCab3.0 v103).

The Mo/Ma subset was reanalyzed independently to better resolve putative cell subtypes. Unsupervised graph-based clustering identified seven distinct Mo/Ma clusters (**Figure 4A**). Some of the gene expression patterns used for cell subtype assignment are displayed in (**Figure 4C**). The five most upregulated genes in each cluster are shown in (**Figure 4D**). The DEGs list for each cluster can be found in (**Supplementary Table 4**). Mo/Ma 6 represented dead or dying cells, based on a low RNA and feature count (**Supplementary Figure 3**).

#### 3.2.1 Alveolar macrophages

Clusters Mo/Ma 0, Mo/Ma 1, Mo/Ma 3 and Mo/Ma 5 overexpressed the macrophage-specific markers *MARCO*, *APOE*, *MSR1* and *CD163*, indicating that these cells were mature alveolar macrophages (AM). Mo/Ma 0 and Mo/Ma 1 had the highest expression levels for *MARCO* and *APOE*. These cells also overexpressed complement genes (e.g. *C1QB*, *C4BPA*, *C1QC* and *C1QA*), suggesting these were activated AMs (24).

Mo/Ma 0 overexpressed *OLFM4*, *FAPB5* and *ANXA2*. This cell cluster was annotated as *FABP5* ^high^ AMs, in reference to the previously described *FABP4* ^high^ (25) or *FABP4* ^+^ (4) AM populations.

Mo/Ma 1 expressed high levels of *FCN1* and *ORM2* (*LOC100050100)*. Transcripts coding for lipid mediators (*ALOX5AP*, *PLTP*, *LTA4H* and *APOE*) were overrepresented. This cell cluster was annotated as *FCN1* ^high^ AMs in line with a previously published classification (4, 25).

Mo/Ma 3 had somewhat lower levels of AM specific markers compared to Mo/Ma 0 and Mo/Ma 1, perhaps indicating that this cell cluster was at an earlier differentiation stage. This cell cluster was characterized by upregulation of *CD5L.* Numerous anti-oxidant genes were also upregulated (e.g. *FTH1*/*LOC111767398*, *GSTP1*/*LOC100059533*, *TXN*, *SOD2* and *SRXN1*). We detected upregulation of *MARCKSL1*, involved in macrophage migration (26). This cell cluster was annotated as *CD5L* ^+^ AMs.

The expression profile of Mo/Ma 5 was dominated by mitosis-associated genes (e.g. *CENPF*, *MKI67*, *TOP2A* and *TPX2*). This cluster was therefore annotated as proliferating AMs (**Figure 4C**).

#### 3.2.2 Monocytes

Mo/Ma 2 and Mo/Ma 4 showed an overall low expression of AM markers. In particular, *APOE*, *MARCO*, *CD163* and complement genes were downregulated compared to the other Mo/Ma clusters. These cells were thus labeled as monocytes. Both Mo/Ma 2 and Mo/Ma 4 expressed high levels of MHCII-associated genes and downregulated *CD16* (**Figure 4C**).

Mo/Ma 2 overexpressed genes associated with macrophage chemotaxis (*SPP1* and *CCL15*) and cell migration (*CD44*, *MMP9*) (27–29). We hypothesized that these cells were blood-derived intermediate monocytes, analogous to the extravascular *CD14* ^+^ *CD16* ^-^ HLA-DR^high^ monocytes described by Evren and colleagues (30). The human classical monocyte marker *CD14* was not upregulated in Mo/Ma 2, but it was overall sparsely expressed in our dataset. We annotated Mo/Ma 2 as intermediate monocytes.

The monocytes Mo/Ma 4 upregulated *PLAC8*, considered a signature gene for patrolling monocytes (31). Most importantly, we detected a clear T cell signature in this cluster (*CD2*, *CD3D, CD3E, CD3G*, *CD5* and *CD7* expression) (**Figure 4C**). Mo/Ma 4 presented an expression profile “halfway” between Mo/Ma2 (intermediate monocytes) and T cells (**Supplementary Figure 5**). Several genes responsible for lymphocyte activation (e.g. *CTSW*, *PTPRCAP* and *LTB*) were also overexpressed. A subpopulation of Mo/Ma 4 overexpressed *LOC100630729.* This gene codes for the immunoglobulin kappa variable 2-30-like protein (**Figure 4C**) expressed in plasma cells. Mo/Ma 4 was annotated as double positive monocyte-lymphocyte cells.

After exclusion of the dead cells (Mo/Ma 6), monocytes (Mo/Ma 2 and Mo/Ma 4), AMs (Mo/Ma 0, Mo/Ma 1, Mo/Ma 3) and proliferating AMs (Mo/Ma 5) represented 30.9%, 64.4% and 4.7% of the Mo/Ma population. While the proportion of monocytes and proliferating AMs was fairly similar among the horses, the distribution of the three distinct AM populations showed a high interindividual variability (**Figure 4B**).

### 3.3 Composition of the T cell population

**Figure 5:**
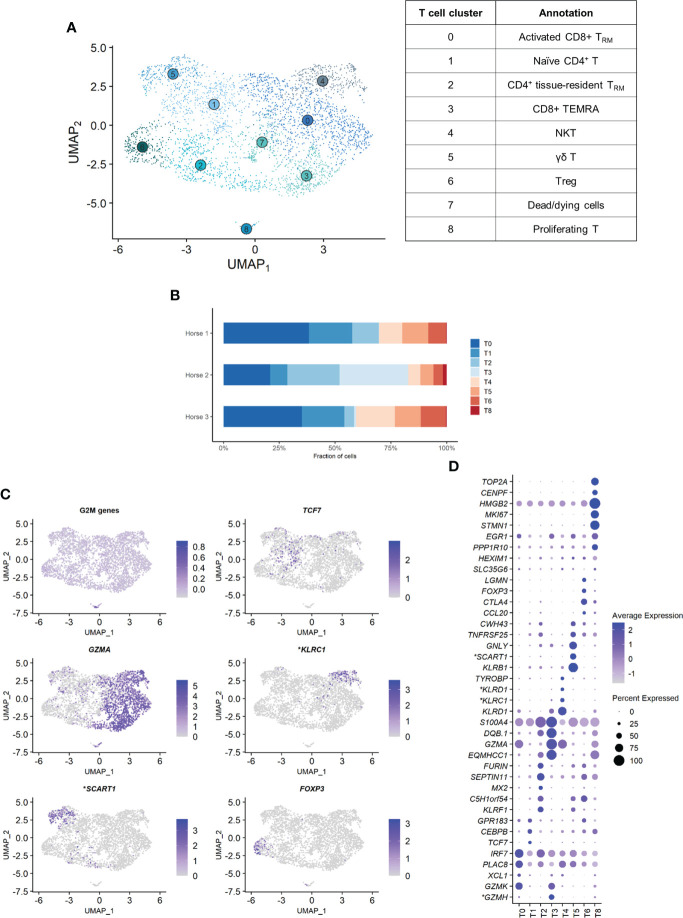
Independent analysis of the T cell population (n=3,465 cells) **(A)** UMAP visualization of the 9 T cell clusters identified, with suggested cluster annotation. *T_RM,_ tissue-resident memory.* **(B)** Distribution of the T cell clusters for each horse, based on scRNA-seq analysis. **(C)** Gene expression patterns used for cell subtype assignment. *LOC100062846 annotated as killer cell lectin like receptor C1 (KLRC1), LOC100066851 as scavenger receptor family member expressed on T cells 1 (SCART1) (NCBI EqCab3.0 v103). **(D)** Dot plot of the 5 most upregulated genes in each cluster (one ribosomal RNA gene and two ribosomal protein genes removed). Dot size is proportional to the percentage of cells expressing the gene. Dot color intensity represents average gene expression. T7 (dead cells) is excluded. *LOC100066851 annotated as SCART1, LOC101910264 as killer cell lectin like receptor D1 (KLRD1), both LOC100062823 and LOC100062846 as KLRC1, both LOC100051986 and LOC100147522 as granzyme H (GZMH) (NCBI EqCab3.0 v103).

Independent reanalysis of the T cell population identified nine clusters (**Figure 5A**). Some of the gene expression patterns used for cell subtype assignment are displayed in (**Figure 5C**). The five most upregulated genes in each cluster are shown in (**Figure 5D**). The DEGs list for each cluster can be found in (**Supplementary Table 5**). T7 constituted dead or dying cells, based on a low RNA count and upregulation of mitochondrial genes (**Supplementary Figure 3**).

Clusters T1, T2 and T6 were CD4^+^ T cells (T helpers), as shown by upregulation of the *CD4* gene and downregulation of the *CD8a* and *CD8b* genes. The remaining clusters T0, T3, T4 and T5 were CD8^+^ T cells (cytotoxic T cells) based on the upregulation of the *CD8a* and/or *CD8b* genes and the downregulation of the *CD4* gene (**Supplementary Figure 4**). The tissue resident marker *ITGAE* was upregulated in the T cell clusters 0, 2, 3 and 6.

T1 represented naïve CD4^+^ T cells, based on the overexpression of *TCF7* (32, 33). Transcripts coding for ribosomal proteins (RP) were overrepresented. Expression of RP genes was not correlated with the cell cycle state. Increased ribosome biogenesis could instead be explained by the high requirement for protein synthesis during cell differentiation in a naïve cell population as previously observed in stem cells (34).

T2 showed high levels of the tissue resident marker *ITGAE* (33) and the T helper marker *CD40LG*. Upregulation of *DUSP1* and *ANXA1* were consistent with antigen-experienced, activated T cells (35, 36). T2 was thus annotated as CD4^+^ tissue-resident memory (T_RM_) cells. Interestingly, the top differentially expressed gene was *KLRF1*, a cytotoxicity regulator whose expression is associated with exhaustion of human memory CD4^+^ T cells (37). *RGS1*, a marker of exhaustion in CD8^+^ T cells (38), was also upregulated in this cluster.

Cluster T6 overexpressed Treg cells canonical markers *FOXP3*, *CTLA4* and *IL2RA* (33, 39) and key transcription factors *FOXO1* (40), *MAF* (41, 42) and *FOXP1* (43), as well as the Treg differentiation regulator *CD27* (44). These cells were annotated as Treg cells.

The expression profiles of the CD8^+^ clusters T0 and T3 were very alike. Both overexpressed the tissue-resident marker *ITGAE* (33), the cytotoxicity effectors *PRF1* and *CTSW*, the granzyme genes *GZMA*, *GZMK* and *GZMH*, and *EOMES* (45), suggesting they were T_RM_ CD8^+^ T cells. One substantial difference between the two clusters was the upregulation of the IFN-stimulated genes *IFI6* and *IRF7* and other genes involved in antiviral response (*PLAC8* and *LY6E*) in T0 (46, 47). These genes code for proteins involved in response to viral infection and are upregulated in activated T cells. The chemokines *CCL4* and *CCL5*, secreted by pathogen-specific effector T cells (48), were also upregulated. We consequently annotated T0 as activated CD8^+^ T_RM_. On the other hand, T3 was characterized by overexpression of tissue-intrinsic genes participating in cytoskeletal structure (*VIM*, *TUBA1A*) and modulation (*S100A4*, *ANXA2*, *EZR*), cell-cell or cell-matrix interactions (*LGALS1, LGALS3*), and membrane scaffolding (*ITM2B*) (33). Additionally, RP genes were downregulated in this cluster, suggesting it was composed of fully differentiated, tissue-adapted cells (49). The DEGs for T3 included *PRF1*, *NKG7*, *HOPX*, *LAG3* and *CCL4* (33), as well as MHCII-associated genes, consistent with activated T cells. We suspected that T3 represented CD8^+^ terminally differentiated effector cells (T_EMRA_) based on the upregulation of *PTPRC* (*CD45*). However, PTPRC average expression was overall low in the cluster and, in absence of PTRPC isoform data in horses, this annotation could not be ascertained. T3 was therefore annotated as MHCII^high^ CD8^+^ T_RM_ cells.

The ubiquitous expression of the T cell markers *CD2*, *CD3* and *NCAM1* indicated that our dataset did not contain true NK cells. However, T4 still demonstrated NK-specific features. Top upregulated genes included genes from the *NKG2* family (*KLRC1*, *KLRK1*) and the related *KLRD1*, *CD160* and *NKG7* genes, all associated with NK function (**Figures 5C, D**). *TYROBP* was also overexpressed, in accordance with previous description of human peripheral NKT cells (50). We thus annotated T4 as NKT cells (*CD1d*-restricted invariant natural killer T cells).

Cluster T5 was annotated as γδ T cells. The second most strongly upregulated gene in this cluster was *LOC100066851*, coding for a *SCART1*-like protein (**Figures 5C, D**). SCARTs are surface receptors found primarily on γδ T cells (51, 52). This cluster shared many DEGs with the equine PBMC cluster annotated as γδ T cells by Patel and colleagues (53). Of note, our BALF γδ T cells also overexpressed genes associated with cytotoxicity such as *KLRB1*, *GNLY* or *KLRF1*.

Cluster T8 was annotated as proliferating T cells based on the high levels of mitosis marker (e.g. *CENP*, *HMGB2*, *TOP2A*) and other markers of cell proliferation such as *MKI67* (**Figures 5C, D**). This cluster comprised both CD4^+^CD8^-^ and CD4^-^CD8^+^ T cells.

The distribution of the nine T cell clusters was comparable in horses 1 and 3. Horse 2 presented a higher proportion of CD4^+^ T_RM_ (T2), MHCII^high^ CD8^+^ T_RM_ (T3, most markedly), and proliferating T cells (T8) (**Figure 5B**).

### 3.4 Composition of the B/plasma cell population

**Figure 6:**
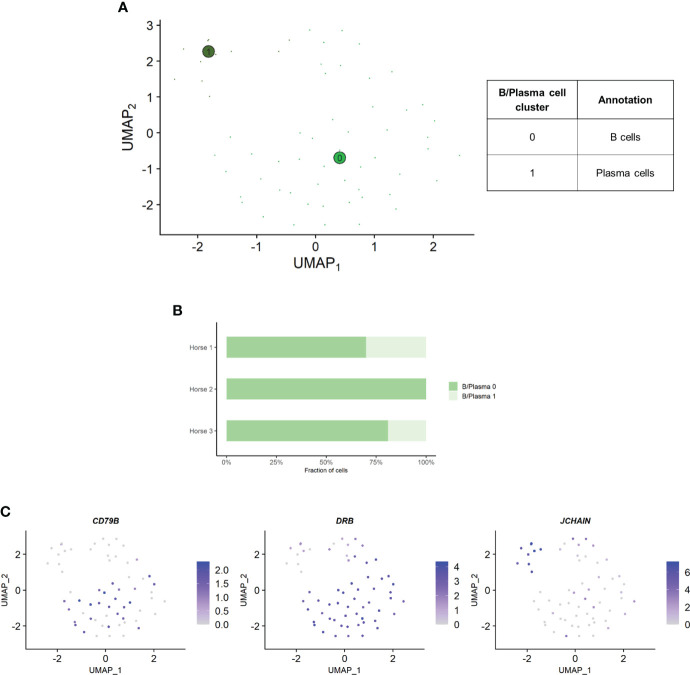
Independent analysis of the B/plasma cell population (n=62 cells) **(A)** UMAP visualization of the two B/plasma cell clusters identified, with suggested cluster annotation. **(B)** Distribution of the B/plasma cell clusters for each horse, based on scRNA-seq analysis. **(C)** Gene expression patterns used for cell subtype assignment. *LOC100147624 annotated as derlin 3 (DERL3) (NCBI EqCab3.0 v103).

Independent analysis of the B/plasma cell population revealed two distinct cell clusters: B/plasma cell 0 and B/plasma cell 1 and B/plasma cell 1 (**Figure 6A**). Some of the gene expression patterns used for cell subtype assignment are displayed in (**Figure 6C**). The DEGs list for each cluster can be found in (**Supplementary Table 6**). B/plasma 1 overexpressed several genes associated with humoral response, some of which being upregulated in equine blood antibody-secreting B cells (*DERL3*, *HSP90B1*, *PPIB* and *SSR3*) (53). *JCHAIN*, coding for the joining chain of multimeric IgA and IgM, was also overexpressed. B/plasma 1 was thus annotated as plasma cells. On the other hand, B/plasma 0 was characterized by high expression of the canonical B lymphocyte markers *CCR1*, *CD74*, *BANK1*, *MS4A1* and *CD79B*. This cluster was annotated as B cells. The two top upregulated genes for B cells were the MHC class II components *DRB (*ortholog of human *HLA-DRB1)* and *CD74*. MHCII expression is lost during differentiation to plasma cells, further supporting our annotation (54). The relative distribution of B and plasma cells differed between horses, with no plasma cells detected in horse 2 (**Figure 6B**).

## 4 Discussion

Our proof of concept single-cell analysis of equine bronchoalveolar cells allowed the identification of the major immune cell populations present in the BALF of adult horses without the need for conventional microscopic cytology or labeled antibodies. We were able to distinguish transcriptionally distinct Mo/Ma and T cell subpopulations relevant for the characterization of different types of immune responses. An unexpected but important finding was the presence of cells or cell pairs expressing both lymphocyte and monocyte markers.

To the best of our knowledge, this is the first scRNA-seq experiment on cryopreserved BALF cells, not only in horses but in any species. We successfully demonstrated that equine bronchoalveolar cells can undergo cryopreservation at -80°C before scRNA-seq. We were able to detect most of the major immune cell types present in equine BALF, with the exception of eosinophils. This was most likely due to their absence or sparsity in the samples, since they were not found on the cytological preparations either. Of note, we found a significant proportion of neutrophils, cells that are notoriously difficult to detect with scRNA-seq due to their high RNAse content. The addition of RNAse inhibitor during sample processing may have prevented RNA degradation. Detection and correct identification of granulocyte types is important for the characterization of equine asthma phenotypes, highlighting the potential of scRNA-seq to investigate this disease.

The major cell populations were identified with a high level of confidence through the analysis of canonical markers and inspection of the top DEGs for each cluster. The analysis of the cell subtypes was more difficult, with their annotation being subject to interpretation. Automated annotation using SingleR with three different reference datasets [the Human Primary Cell Atlas, a human lung cell scRNA-seq dataset (55) and a ferret BALF cell dataset (14)] did not improve the annotation of cell subtypes (data not shown). ScRNA-seq experiments have introduced unprecedented levels of complexity to the classification of cells, challenging the traditional definition of a cell type. Cell types can be defined with various criteria, including their phenotypes, lineages and states (56). Microscopy can only identify a few cell populations based on their morphologic features. On the other hand, use of labeled antibody techniques allow the exploration of cell types at a much higher resolution. These methods, however, rely on the presence or absence of a cell surface marker, shaping our definition of what is a cell type. Additionally, the range of cell types that can be identified in horses is hampered by the limited pool of available equine-specific antibodies. With scRNA-seq, we can appreciate various degrees of expression of a plethora of marker genes, blurring the lines between transcriptionally similar cell populations. The different layers formed by the lineage origin, the differentiation stage, or the activation state can be difficult to untangle. The interesting concept of a cell periodic table was recently proposed (57). Such a table could reconciliate the various strati detected with single-cell analysis to redefine cell types and states. Our dataset could contribute to the assembly of such a periodic cell type table for horses. It will also aid in the construction of an equine lung atlas, similar to the human lung atlas initiative^b^.

We observed a marked discrepancy of the lymphocyte/macrophage ratio between cytology and scRNA-seq. This resulted from differences between the techniques used for counting, since the cytological DCCs before and after sample cryopreservation were comparable. We analyzed the data provided by Fastrès et al. and noticed that the scRNA-seq analysis of healthy dogs’ BALF cells was similarly biased toward a higher lymphocyte count compared to cytology (15). The lymphocyte/macrophage ratio was five times higher with scRNA-seq, akin to our findings. The droplet-based sequencing method could theoretically bias the observed cell distribution through preferential selection of smaller cells. To our knowledge, this has never been reported in immune cells. Another potential explanation for the lower macrophage proportion is the high production of RNAses by granulocytes, leading to greater mRNA degradation and thus lower transcript recovery (58). In such cases however, we would have expected no or low detection of neutrophils and mast cells with scRNA-seq. An alternative and more plausible hypothesis is that conventional cytological DCC diverges stronger from the biological reality than the scRNA-seq based classification. Studies comparing flow cytometry with manual counting have revealed that conventional cytology led to lymphocyte count underestimation (59) or macrophage count overestimation (60). Explanations put forward were the limited number of cells counted under light microscopy, inaccurate classification due to apoptotic changes and uneven cellular distribution on cytological preparations. Indeed, cytocentrifugation tends to propel the lymphocytes to the margins of cytological slides, leading to their underestimation during manual counting (61). Furthermore, in our experience, single epithelial cells can be mistaken for macrophages in light microscopy, potentially leading to an overestimation of the latter in the DCC. In summary, the lymphocyte/macrophage ratio in our cell population was markedly higher with scRNA-seq compared to cytology. Whether scRNA-seq or cytological cell distribution best reflects the biological reality remains to be determined. Since current guidelines to characterize lower airway inflammation in horses are based on cytological DCC (12), we recommend that cell distribution is concomitantly assessed *via* conventional cytology when performing scRNA-seq on equine BALF cells.

Independent analysis of the Mo/Ma cluster enabled a clear distinction between monocytes and AMs based on the expression of a selection of macrophage-specific genes. Previous scRNA-seq studies on BALF from other species (14, 55) and on equine peripheral blood (53) used *CD14* to identify monocytes. The expression of this canonical marker was barely detectable in our dataset, similar to previous observations in dog BALF (62). Yet, equine BALF monocytes have been described as CD14^+^ cells based on flow cytometric data (63). We suspect that *CD14* expression was blunted by the higher expression of other genes in our cell population. The major DEGs for the three distinct quiescent AM clusters (Mo/Ma 0, 1 and 3) were mostly associated with regulation of inflammation.Cluster Mo/Ma 0 (*FABP5* ^high^ AMs) overexpressed *OLFM4*, *FAPB5* and *ANXA2*. *OLFM4*, a marker of severe lung disease, may be involved in the inflammatory response regulation (64, 65). *FABP5* has anti-inflammatory properties in allergic lung inflammation (66). *ANXA2* negatively regulates TLR4-triggered inflammatory responses, thus preventing excessive inflammation (67). Moreover, the myeloid cell-derived proteins *S100A8* and *S100A9*, involved in lung protective mechanisms (68), were also upregulated in this cluster. The concurrent downregulation of MHCII-related genes in Mo/Ma 0 further supports an anti-inflammatory phenotype (69). Cluster Mo/Ma 1 (*FCN1* ^high^ AMs) was characterized by upregulation of the *FCN1* gene coding for ficolin 1, a pattern-recognition receptor involved in innate immunity. This cluster also expressed high levels of *ORM2* (*LOC100050100*), an acute-phase protein preventing inflammation in adipose and neural tissue (70, 71). Mo/Ma 3 (*CD5L* ^+^ AMs) had somewhat lower levels of AM specific markers compared to Mo/Ma 0 and Mo/Ma 1, perhaps indicating that this cell cluster was at an earlier differentiation stage. We detected upregulation of *MARCKSL1*, which plays a crucial role in macrophage migration (26). Mo/Ma 3 could thus represent recently migrated AMs. This cell cluster also overexpressed *CD5L*, which promotes M2 macrophage polarization (72, 73). However, none of the clusters displayed a clear M1 or M2 phenotype. AM phenotypes deviating from the classic M1 or M2 phenotypes have already been identified with flow cytometry (74) and RNA-sequencing (75). It has been suggested that M1 and M2 actually represent extremes of a polarization state continuum, rather than stable phenotypes (76, 77). Similar to our findings, previous BALF scRNA-seq studies on ferrets (14) and dogs (15, 62) detected transcriptionally distinct AM clusters. These may represent discrete cell subtypes or different activation states of the same cell type.

We annotated Mo/Ma 2 as intermediate monocytes in reference to a recently published developmental map of human lung macrophages (30). In this study, HLA-DR^high^ CD14^+^CD16^-^ monocytes egressing from peripheral blood were shown to be AM precursors. Until recently, it was assumed that the pool of AMs was mostly maintained through local self-renewal, with minimal contribution of blood-derived monocytes [reviewed in (78)]. While initial studies were performed in mice, latter work in healthy humans suggests that human AMs are mostly derived from circulating monocytes (78). This discrepancy could stem from the constant exposure of humans to inhaled antigens, in contrast to the germ-free environment of laboratory mice (78). Our findings support the idea that equine AMs mostly arise from the differentiation of peripherally-derived monocytes, similar to what is described in humans. This may reflect a constant renewal of AMs to adapt the lung immune response to the dynamic antigenic load and to the non-specific irritants horses are exposed to *via* the inhalation route in their typical stable environments (12). Single-cell analysis of lung cells collected at sequential developmental stages in the horse may definitely elucidate the origin of equine AMs.

Cluster Mo/Ma 4 stood out by the co-expression of lymphocyte and monocyte markers. Similar double positive cells were detected in three other BALF scRNA-seq studies (4, 30, 62). This was attributed to either ambient RNA contamination (30, 62) or to doublet formation (4). We considered several different hypotheses to explain this unusual expression pattern, including engulfing macrophages, a novel dual lineage cell population, technical multiplets (capture of several cells in a single droplet) and immune cell complexes. The engulfing macrophages hypothesis was not convincing. First, we assume these cells are monocytes based on downregulated macrophage-specific genes, including those required for phagocytosis (e.g. *CD163*) *(* 79). Second, we did not detect markers specific for neutrophils, the cells that are most likely to be phagocytosed by macrophages (80). While the presence of a novel dual lineage cell population expressing both lymphocyte and monocyte markers cannot be definitely dismissed, it seems unlikely that several flow cytometry-based studies on equine BALF (59, 63, 81, 82) would have failed to detect them. Lymphocytes and monocytes originate from separate cell lineages, namely lymphoid and myeloid. A new dual lineage cell type would thus call into question our current understanding of cell ontology. Mo/Ma 4 could represent technical doublets and/or multiplets. However, this does not explain why only lymphocytes and monocytes signatures are combined, and not signatures for other cell types. Indeed, encapsulation of two or more cells within a droplet during libraries generation should be random. Therefore we propose that the Mo/Ma 4 cluster represents mostly monocyte-T cell complexes, as recently described in human peripheral blood (83, 84). We hypothesize that the Mo/Ma 4 subset overexpressing the Igκ-like protein gene corresponds to monocyte-B cell complexes. Immunological stimuli such as immunization or disease affect the frequency and the phenotype of immune cell complexes (83). Studying their formation and specificity may help unveil the underlying mechanisms of specific equine respiratory conditions such as equine asthma. To determine whether monocyte-lymphocyte complexes are truly present in equine BALF, we suggest combining scRNA-seq with imaging flow cytometry.

We found the annotation of T cell subsets challenging. Similarly to Patel and colleagues working with equine PBMCs (53), we could not identify the Th1, Th2 and Th17 phenotypes, as their specific chemokines were only sparsely expressed in our dataset. Assigning a cell identity to CD8^+^ clusters was also difficult, probably because gene expression changes in a linear fashion throughout differentiation (45, 85). Further single-cell studies and replication experiments should help to better delineate the transcriptional signatures of the different T cell populations. We annotated one of the T cell cluster as NKT cells. The existence of NKT cells in horses was already reported (86, 87). While NKT cells are well characterized in humans and mice, their transcriptional signature and pattern of surface receptors remain to be defined in horses. A cell cluster with an expression profile similar to our NKT cell population was detected using scRNA-seq on equine PBMCs, but was annotated as NK cells despite being CD3^+^ (53).Genes coding for RP were overexpressed in the two lymphocyte cell populations (T and B/plasma cells), a common observation in scRNA-seq of lymphocytes. This is likely a consequence of minimal transcriptional activity of most other genes, resulting in an apparent relative increase of abundantly expressed transcripts, such as those encoding RP. This could also reflect the high translational activity required for the cell types respective function, such as cytokine or antibody production (88–90). Even though the DEG list of some T cell clusters were dominated by RP genes, filtering them did not significantly affect cell clustering (data not shown). Moreover, we found that the differential expression of RP genes between T cell clusters corroborated our cluster annotation, with upregulation at the early differentiation stage (CD4^+^ naïve T cells) and downregulation at the terminal differentiation stage (MHCII^high^ CD8^+^ T_RM_) (34, 49).

Three horses from our teaching herd were included in this pilot study out of convenience. These horses were affected with mild-to-moderate equine asthma, but sampled in a phase of clinical remission. Interestingly, horse 2 presented higher proportions of *FABP5* ^high^ AMs, CD4^+^ T_RM_, MHCII^high^ CD8^+^ T_RM_ and proliferating T cells, and no plasma cells. This may reflect intrinsic individual differences of the immune response and/or in the environmental exposures encountered throughout lifetime.

A first pre-processing analysis found a low percentage of reads mapping to transcriptome (mean 33.5%, data not shown). We suspected that this resulted from the poor quality of the horse genome annotation, especially at the untranslated 3’-ends of genes. Therefore, we manually extended the annotations of the 3’-untranslated regions for all transcripts listed in the reference genome by 2 kb, as described elsewhere (91). The mapping to transcriptome substantially improved (mean 53.4%), reaching levels comparable with humane or murine studies. This highlights the need for an improved annotation of the equine reference genome.

While a large amount of new information was gained from this study, we acknowledge some limitations. Our study population was small, due to the high cost of scRNA-seq. The number of cells sequenced was, however, sufficient to gather meaningful initial data. While our ability to detect rare cells (e.g. eosinophils) may have been hindered, we could still demonstrate that equine BALF cells can be successfully used for scRNA-seq after cryopreservation. For the present proof-of-concept study, we sampled BALF from asthmatic horses in remission available on site. The gene expression profiles obtained may thus differ from those of horses free of respiratory diseases. A larger-scale study using healthy horses is required to define a single-cell atlas of equine BALF cells. Most single-cell studies are conducted on fresh samples in order to optimize cell viability and prevent transcriptional modifications associated with sample handling. We wanted to demonstrate the feasibility of cryopreservation before sequencing to facilitate the design of larger, potentially multi-centric single-cell studies. Cryopreservation will allow collection of BALF from different horses on separate days for later batched library preparation and sequencing. Analyzing several samples in one single run minimizes batch effect and significantly reduces sequencing cost. Cryopreservation of cells and tissues have a minimal effect on transcriptional profiles obtained with scRNA-seq (92, 93). However, T cells may be more affected by cold preservation, with populations declining over time and an expression profile biased toward cytotoxicity genes (92, 93). Potential confounding effects of cryopreservation on the expression profiles of specific clusters may have been missed in our dataset due to the currently limited knowledge of cell type-specific transcriptional signatures. The present study was not designed to assess the effect of cryopreservation on gene expression. This should be investigated in future experiments comparing directly fresh and cryopreserved equine BALF samples at the single-cell level. A major limitation of scRNA-seq studies is the difficulty to validate the annotation of cell clusters. Considerable efforts are put into the construction of extensive standardized single-cell atlases (94). Unfortunately, complementary methods that could corroborate the assigned cell clusters (e.g. flow cytometry) fall short in resolution compared to scRNA-seq. The development of new single-cell platforms combining different experimental approaches, such as concurrent gene expression and surface protein labeling, will help to fill this gap.

## 5 Conclusion

Our findings indicate that scRNA-seq technology is applicable to cryopreserved equine BALF cells, enabling the identification of its major immune cell populations. The sample processing protocol developed for this study may be applied to equine BALF cells and thus allow large-scale single-cell sequencing experiments in horses. Here we provide the single-cell gene expression profiles of the bronchoalveolar cells collected from asthmatic horses in remission. Our collection of signature genes will facilitate cell clustering in forthcoming equine scRNA-seq investigations. We anticipate that single-cell transcriptomic studies will generate novel paradigms in equine respiratory research. Importantly, scRNA-seq will be a powerful tool for the identification of equine asthma endotypes.

## Data availability statement

The datasets presented in this study can be found in online repositories. The names of the repository/repositories and accession number(s) can be found below: https://www.ebi.ac.uk/ena, PRJEB51962. The R code used for data analysis is available at https://github.com/vetsuisse-unibe/ScRNAseq-BALF-paper.

## Ethics statement

The animal study was reviewed and approved by Animal Experimentation Committee of the Canton of Bern, Switzerland.

## Author contributions

VJ and VG supervised the study. SS, TL, and VG contributed to the study conception and design. VG acquired funding. SS examined the horses, collected and processed the samples. LP processed and read the cytological preparations. PN coordinated the library preparations and acquired the scRNA-seq data, which was then analyzed by SS and VJ. SS interpreted the data and prepared the original manuscript draft. All authors contributed to manuscript revision, read and approved the submitted version.

## Funding

Swiss National Science Foundation (Grant No. 31003A-162548/1); Internal Research Fund of the Swiss Institute of Equine Medicine, Bern, Switzerland (ISMEquine Research No. 33-890). Open access funding was provided by the University of Bern.

## Acknowledgments

The authors would like to thank med. vet. Michelle Wyler and med. vet. Nicole Altermatt for their assistance in sample collection. We thank the Next Generation Sequencing Platform of the University of Bern for performing the sequencing experiments and the Interfaculty Bioinformatics Unit of the University of Bern for providing the high performance computing infrastructure. The authors furthermore acknowledge the laboratory technicians of the Central Diagnostic Laboratory of the Vetsuisse Faculty for their assistance in sample processing.

## Conflict of interest

The authors declare that the research was conducted in the absence of any commercial or financial relationships that could be construed as a potential conflict of interest.

## Publisher’s note

All claims expressed in this article are solely those of the authors and do not necessarily represent those of their affiliated organizations, or those of the publisher, the editors and the reviewers. Any product that may be evaluated in this article, or claim that may be made by its manufacturer, is not guaranteed or endorsed by the publisher.

## Footnotes

a The Human Protein Atlas. https://www.proteinatlas.org/ [Accessed May 23, 2022]

b The Human Cell Atlas. https://www.humancellatlas.org/ [Accessed May 23, 2022]
