# Erratum: Single-cell gene expression analysis of cryopreserved equine bronchoalveolar cells

**DOI:** 10.3389/fimmu.2023.1178189

**Published:** 2023-03-13

**Authors:** Frontiers Production Office

**Affiliations:** Frontiers Media SA, Lausanne, Switzerland

**Keywords:** single-cell mRNA sequencing, cell cryopreservation, equine respiratory system, equine immunology, cell annotation

An omission to the funding section of the original article was made in error. The following sentence has been added: “Open access funding was provided by the University of Bern”.

The original version of this article has been updated.

