# Supplementary material for: Single-cell gene expression analysis of cryopreserved equine bronchoalveolar cells
Source: Front Immunol. 2022 Aug 23;13:929922. doi: 10.3389/fimmu.2022.929922 (PMC9467276; doi:10.3389/fimmu.2022.929922)
Supplement: Supplementary file 1 [file DataSheet_1.zip › Supplementary Material/Supplementary Figures.docx]

Supplementary Figures


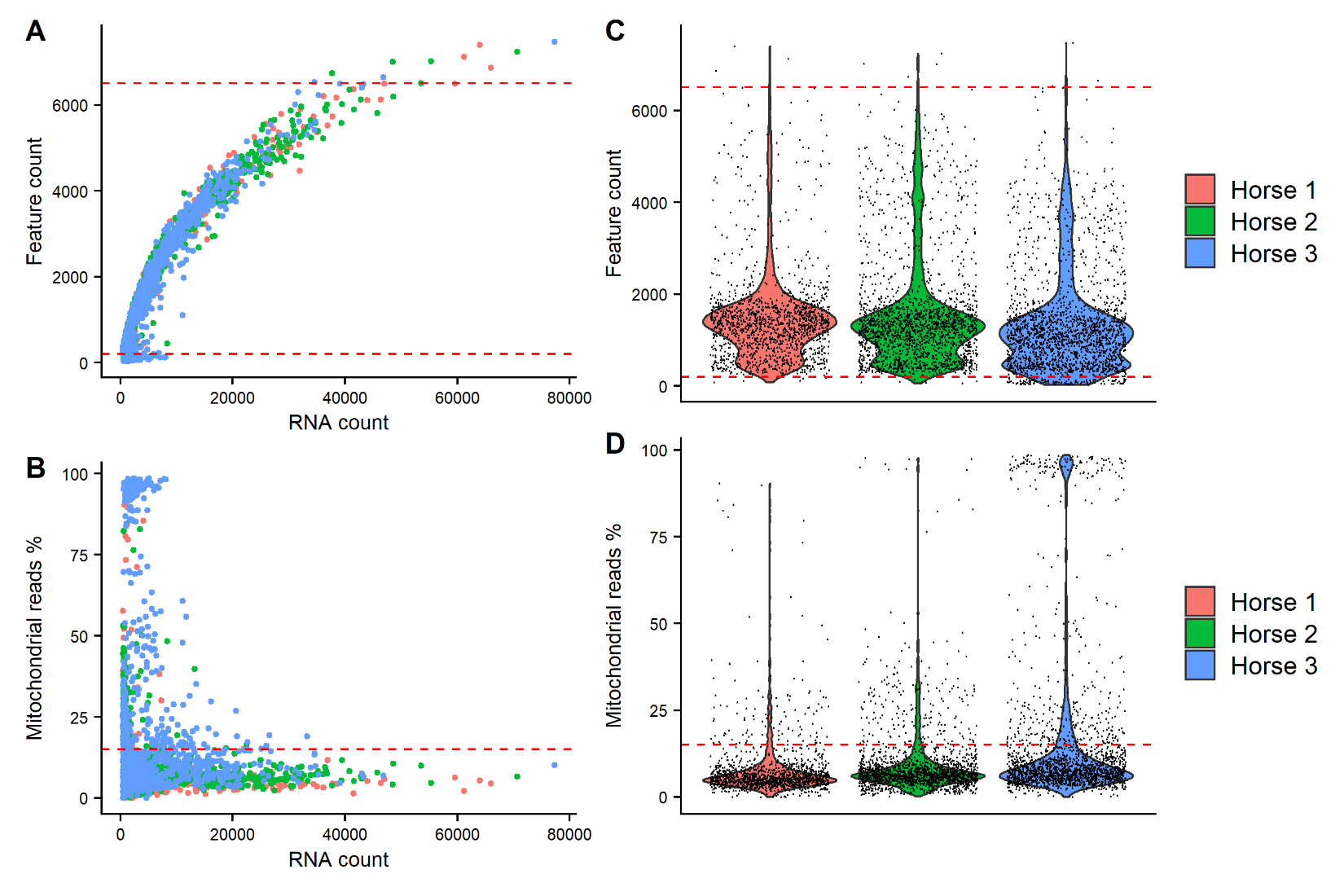


**Supplementary Figure 1.** Quality control analysis performed with the R package Seurat 4.0 on the scRNA-seq data generated from the cryopreserved bronchoalveolar lavage cells of 3 horses (n=5,408 cells). **(A)** Scatter plot showing the relationship between feature count and RNA count. Points are colored according to sample origin. **(B)**  Scatter plot showing the relationship between mitochondrial read expression and RNA count. Points are colored according to sample origin. **(C)** Violin plot showing the feature count in each sample. **(D)** Violin plot showing mitochondrial read expression in each sample. *Filtering thresholds (red dashed lines) were subsequently set to “feature count ≥ 200 or < 6,500”, and “mitochondrial read expression ≤ 15%”.*


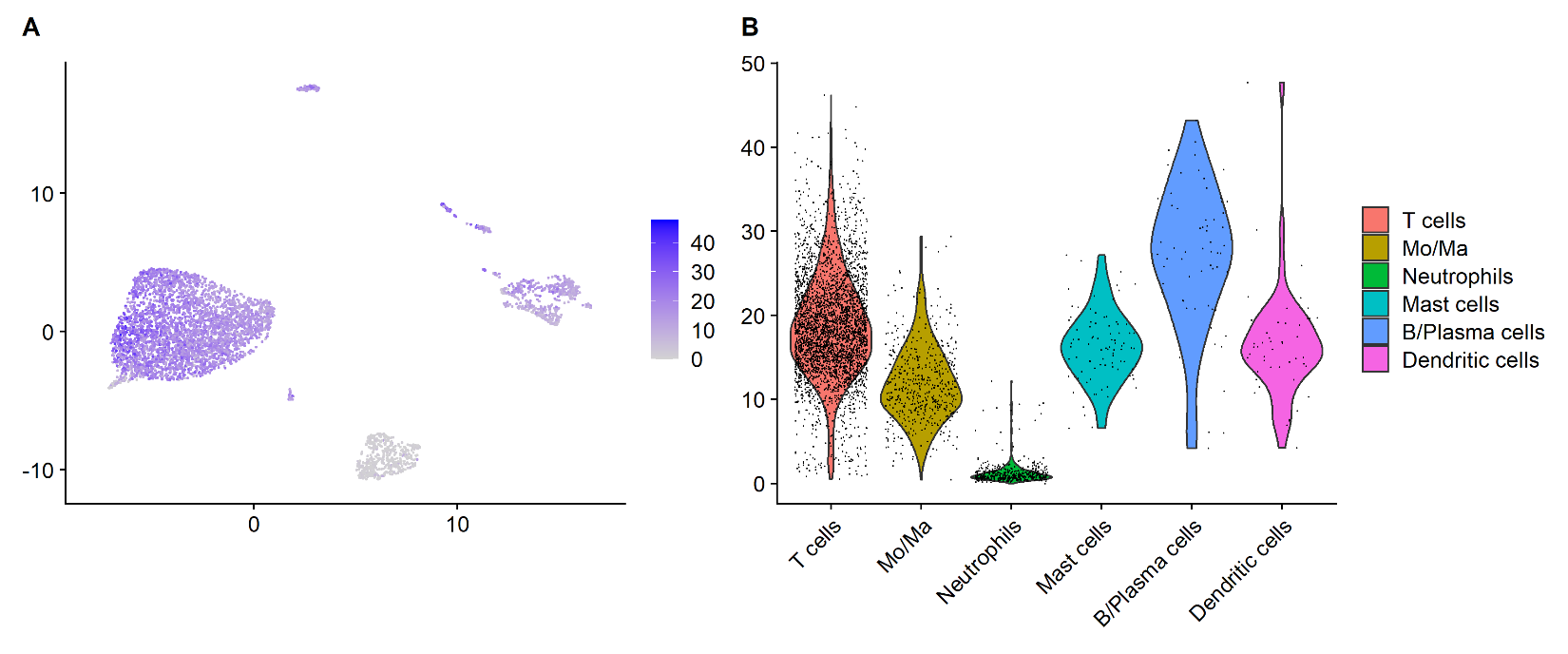
**Supplementary Figure 2.** Ribosomal protein genes differential expression across the six major cell populations (n=4,631 cells) visualized with UMAP (A) and with a violin plot (B).

**
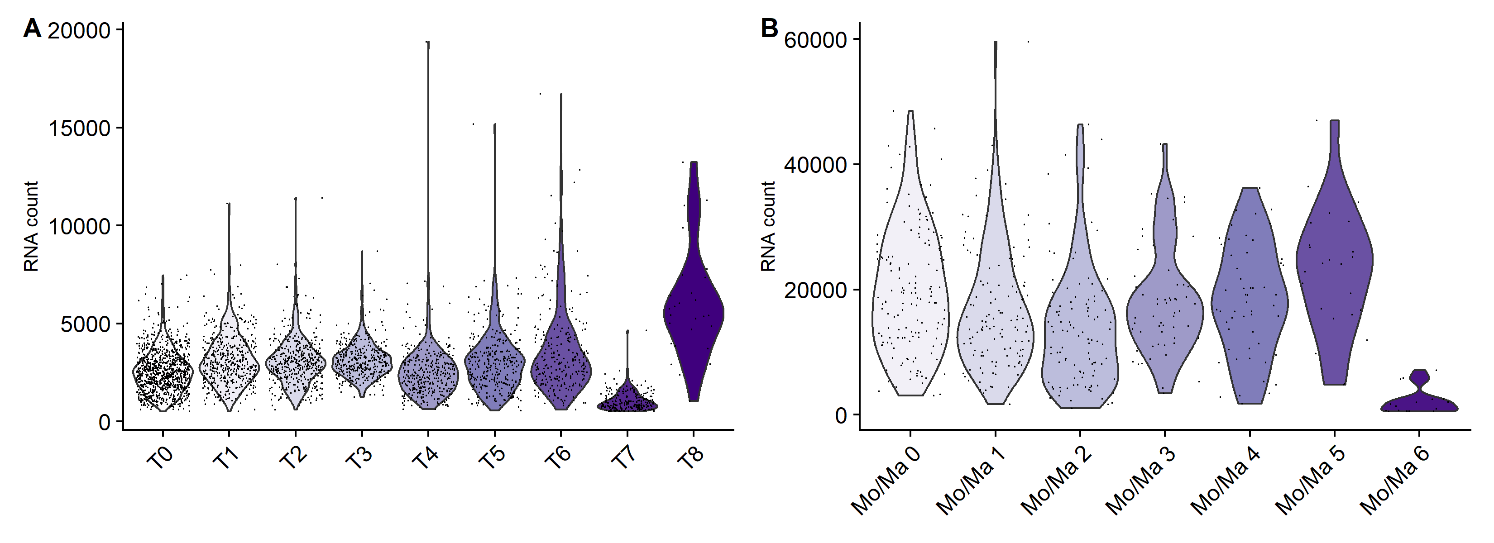
Supplementary Figure 3.** RNA count per Mo/Ma (A) or T cell (B) cluster. The low RNA count in Mo/Ma 6 and T7 indicates dead or dying cells.


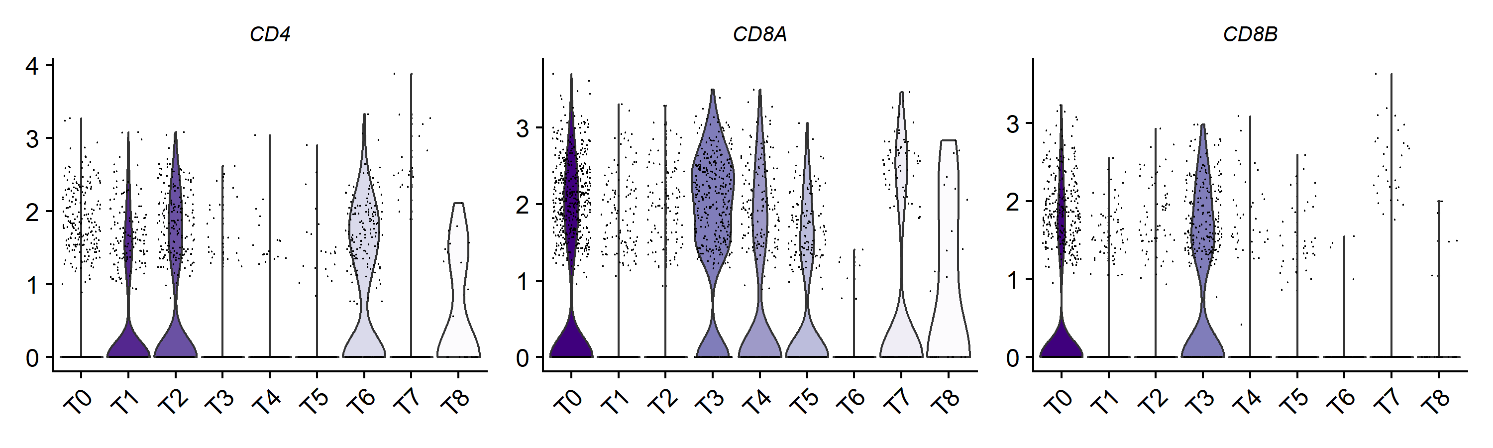
**Supplementary Figure 4.** Expression of CD4, CD8A and CD8B among T cell clusters.

**A**
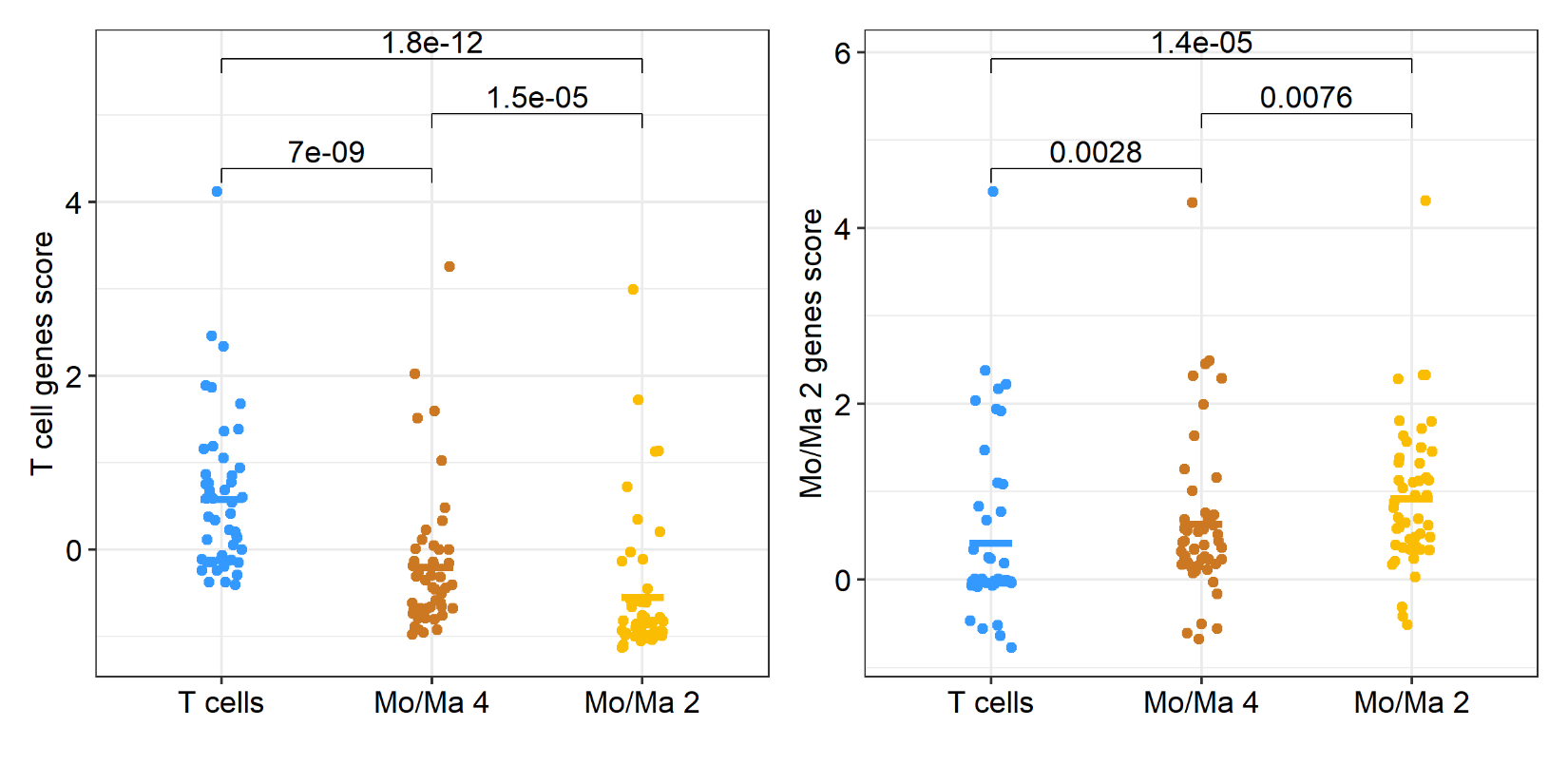


**B**


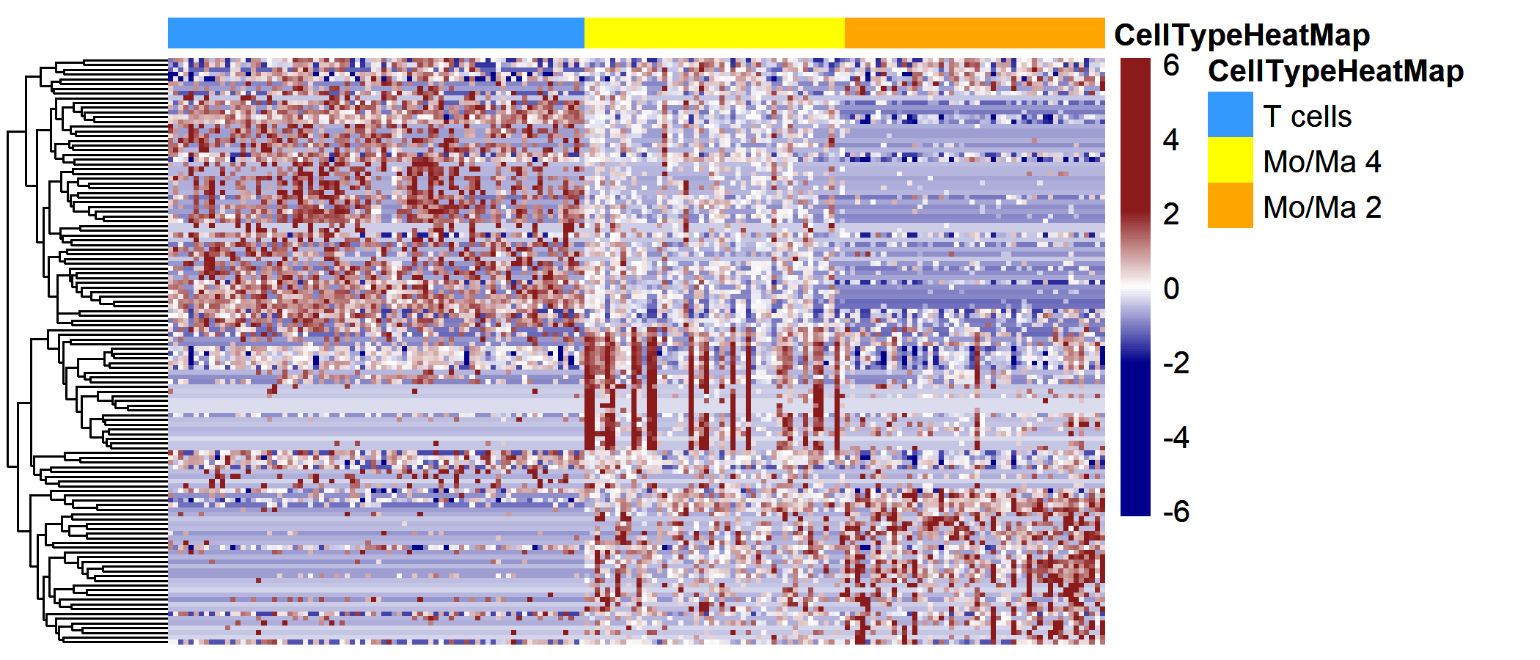


**Supplementary Figure 5:** **Identification of a T cell signature in the monocyte/macrophage cell cluster Mo/Ma 4.** (A) T cell and intermediate monocyte (Mo/Ma 2) gene scores in the T cell, Mo/Ma 2 and Mo/Ma 4 clusters. (B) Heatmap representing single cell gene expression of the 50 most upregulated genes in T cells, intermediate monocytes (Mo/Ma 2) and presumptive monocyte-lymphocyte complexes (Mo/Ma 4).
